# Supplementary material for: Selective packaging of mitochondrial proteins into extracellular vesicles prevents the release of mitochondrial DAMPs
Source: Nat Commun. 2021 Mar 30;12:1971. doi: 10.1038/s41467-021-21984-w (PMC8009912; doi:10.1038/s41467-021-21984-w)
Supplement: Supplementary file 1 — Supplementary Information [file 41467_2021_21984_MOESM1_ESM.pdf]

**Supplementary information**

# Selective packaging of mitochondrial proteins into extracellular vesicles prevents the release of mitochondrial DAMPs

**Todkar et al**

This file contains

**Supplementary Figures (1-4)**
**Supplementary Table 1**

## Supplementary Figure 1

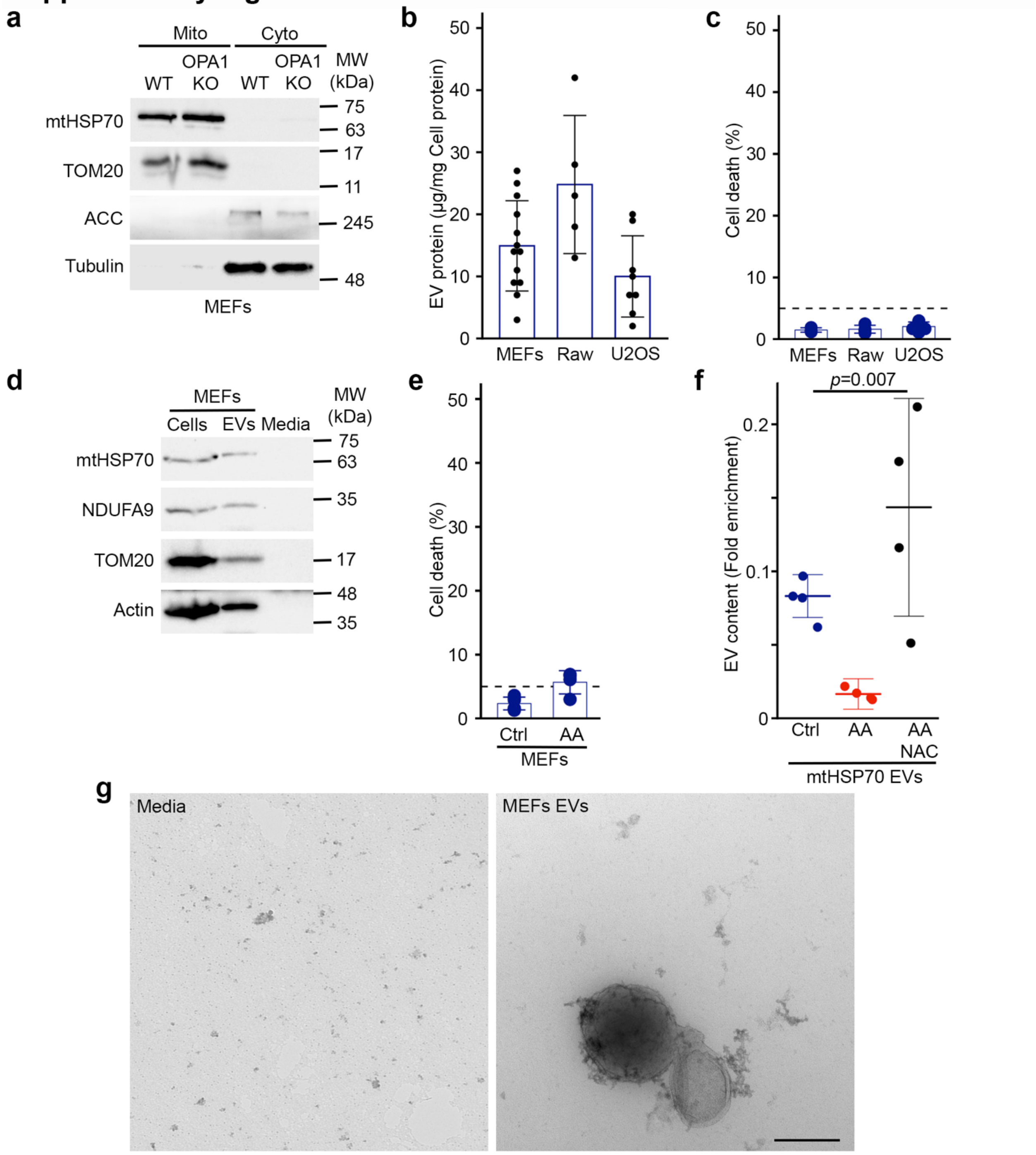


**Supplementary Figure 1. Isolation of mitochondria and EVs.** (A) Isolation of MEFs mitochondria. Mitochondrial and post-mitochondrial fractions were analysed by western blot for mitochondrial (mtHSP70, TOM20) and cytosolic (ACC, Tubulin) markers. (B) Quantification of EV protein yields relative to cellular protein content. Each point represents one experiment (MEFs,

n=13; Raw, n=5; U2OS, n=8). Bars show the average ± SD. (C) Cell viability was measured by Trypan Blue (dashed line represents 5% cell death). Individual points represent independent experiments (n=4). Bars show the average ± SD. (D) Non-conditioned media does not contain mitochondrial proteins. EVs were isolated from non-conditioned media or media exposed to cells and analysed for the presence of mitochondrial proteins and actin. (E) Cell viability (measured by Trypan Blue) following 24 hours treatment of WT MEFs with AA. Individual points represent independent experiments (n=3). The dashed line represents 5% death. Bars show the average ± SD. (F) NAC rescues the inclusion of IM/matrix proteins into EVs of AA-treated cells. EVs were isolated from WT MEFs treated with AA in the absence (Red) or the presence of 10 mM NAC (Black) and the presence of mtHSP70 in EVs measured as in Figure 1. Individual points represent independent experiments (n=4). Bars show the average ± SD. One-way ANOVA. (G) EVs isolated from non-conditioned media as in (D) were analysed by EM. Images representative of 3 independent experiments are shown. Scale bars, 200 nm.

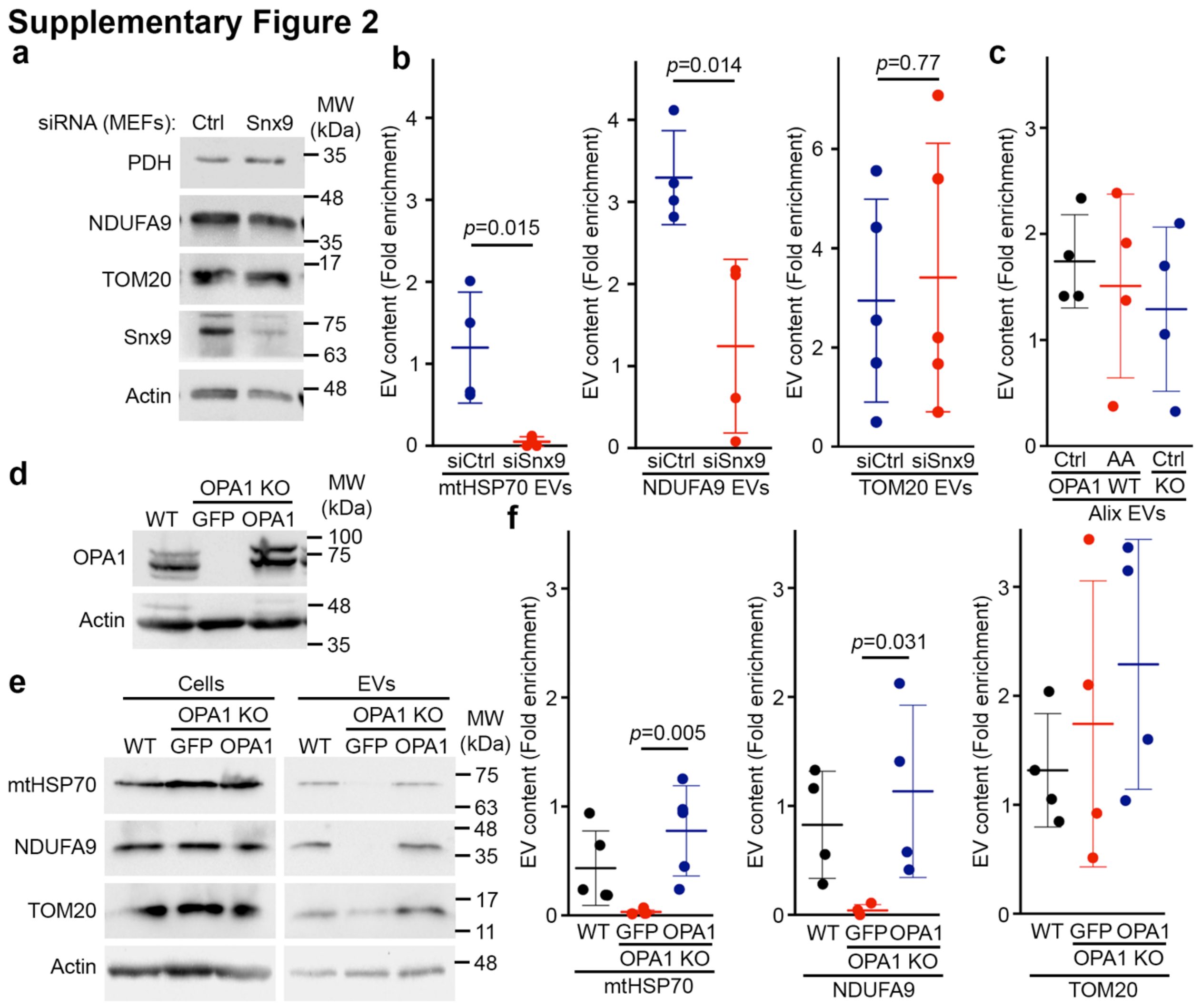


**Supplementary Figure 2. Regulation of mitochondrial EV content by Snx9 and OPA1.** (A) Knockdown of Snx9 (B) Enrichment of the indicated mitochondrial proteins in siCtrl (Blue) or siSnx9 (Red) EVs was measured by western blot as in Figure 1. Individual points represent independent experiments (n=4, except for TOM20 where n=5)). Bars show the average ± SD. Two-sided t-test. (C) The inclusion of the exosomal marker Alix into EVs is not altered following AA treatment or OPA1 deletion. Quantification was done as in as in Figure 1. Individual points represent independent experiments (n=4). Bars show the average ± SD. (D-F) Reintroduction of OPA1 in OPA1 KO MEFs rescues the inclusion of mitochondrial proteins into EVs. EVs from WT MEFs (Blue) and OPA1 KO MEFs stably expressing GFP (Red) or OPA1(Black) were analysed by western blot (OPA1 levels in (D), representative western blot in (E). Data was analysed as in Figure 1 (F). Individual points represent independent experiments (n=4). Bars show the average ± SD. One-way ANOVA.

## Supplementary Figure 3

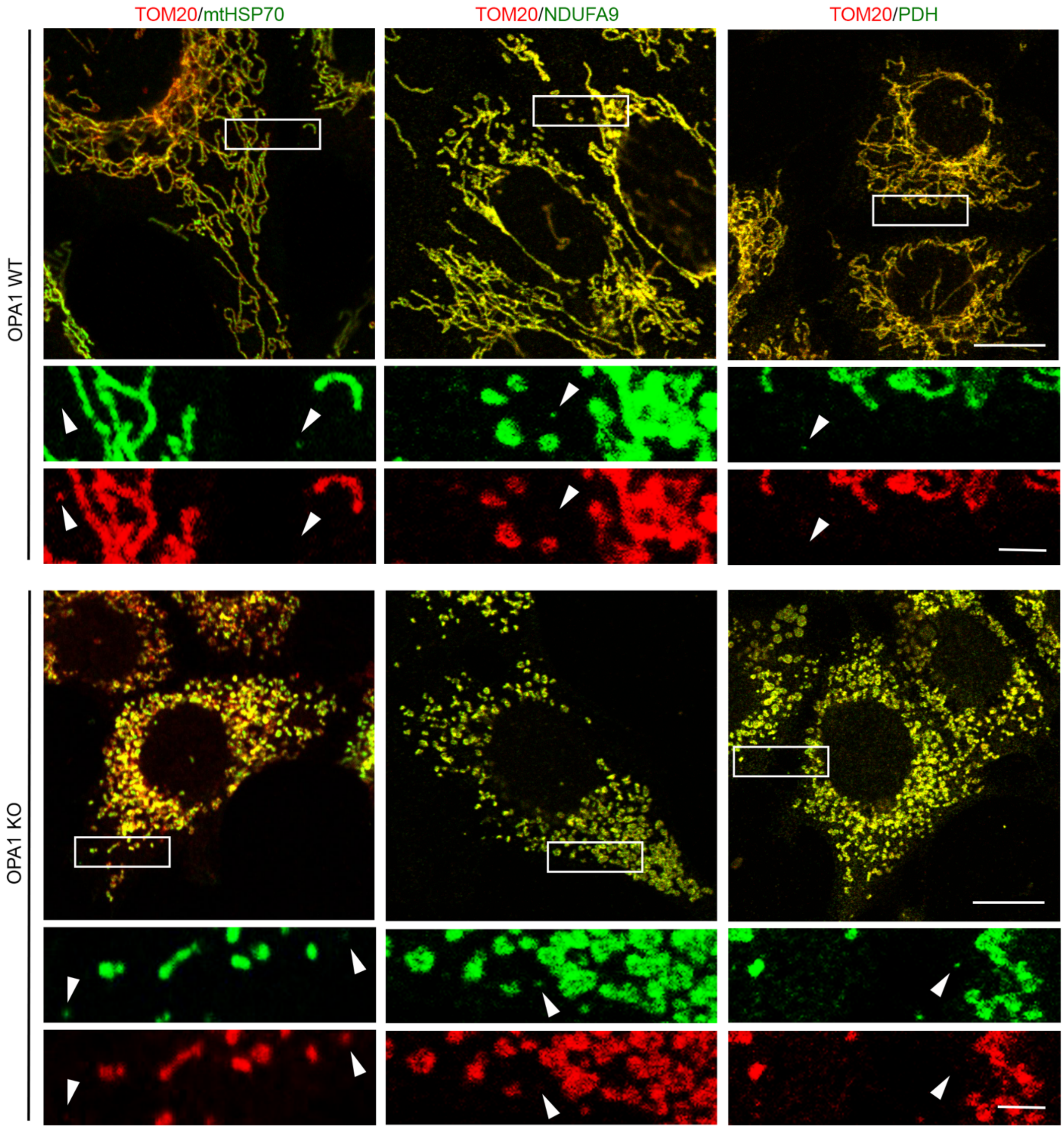


**Supplementary Figure 3. Immunofluorescence data for OPA1 KO MDVs.** WT and OPA1 KO cells were stained with the indicated antibodies and imaged. Representative images are shown. Scale bar, 10 µm for the full image, 2 µm for the insets. Arrows denote MDVs.

## Supplementary Figure 4

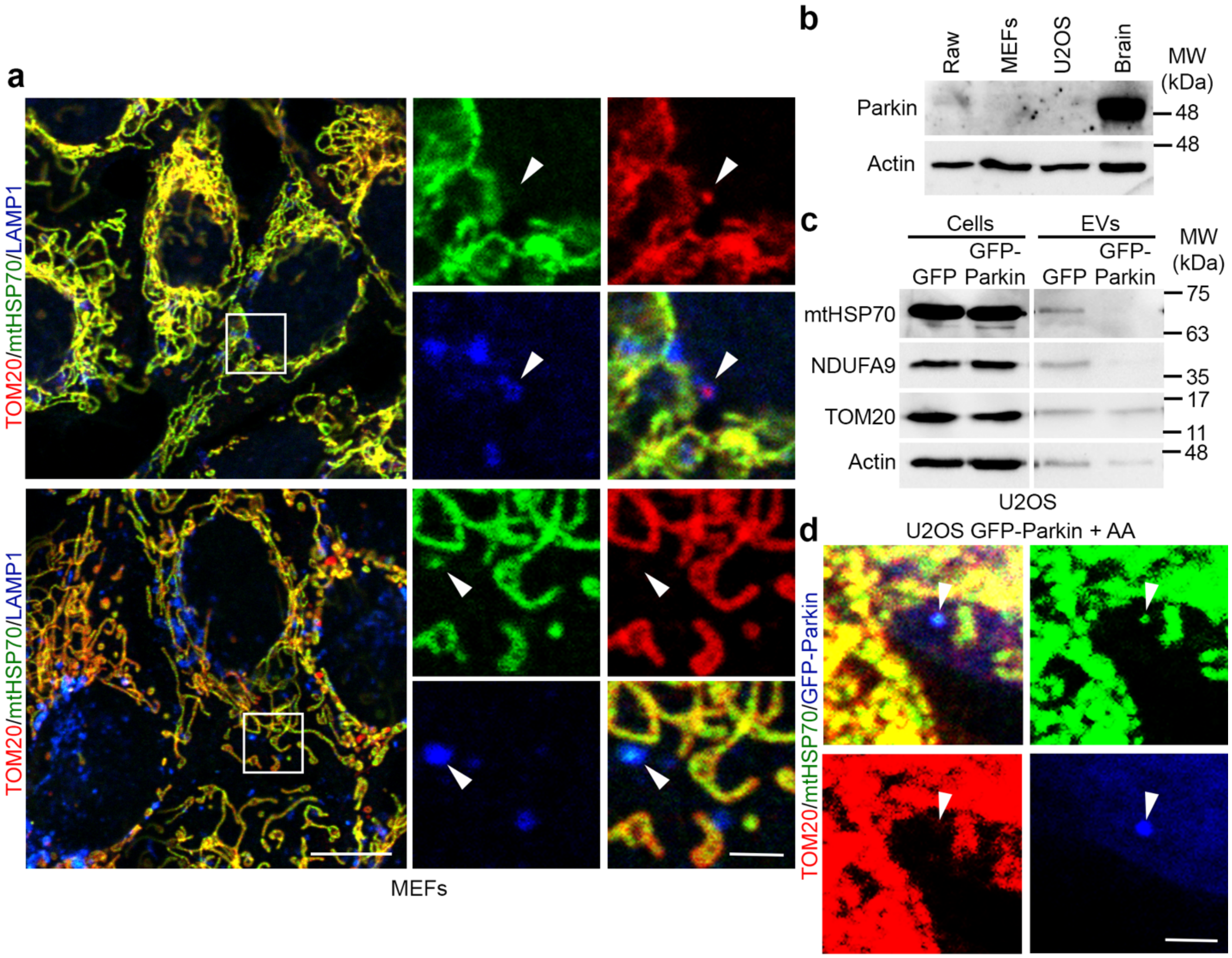


**Supplementary Figure 4. Activation of the lysosomal MDV pathway.** (A) WT cells were stained with antibodies against mitochondrial proteins (TOM20, mtHSP70) and the lysosomal marker LAMP1 and imaged. (A) TOM20-positive MDV (Red) colocalized with LAMP1 (Blue). Scale bar, 10 µm; 2 µm for insets (B) Representative western blot showing the Parkin expression in mouse brain and the cell lines used in this study. (C) Representative western blot showing the decreased inclusion of IM/matrix proteins into EVs of GFP-Parkin expressing U2OS cells. (D) mtHSP70-positive MDV (Green) colocalized with Parkin (Blue). U2OS cells stably transfected with GFP-Parkin were treated with AA and stained for TOM20 (Red) and mtHSP70 (Green). Arrowhead denote a mtHSP70 MDV colocalizing with GFP-Parkin (Blue). Scale bar, 2 µm. Representative images are shown.

## Supplementary table 1

| Gene | Forward Primer | Reverse Primer |
|---|---|---|
| cytochrome *c* oxidase 1 (mouse) | 5'-GCCCCAGATATAGCATTCCC-3' | 5'-GTTCATCCTGTTCCTGCTCC-3’ |
| 18S ribosomal RNA (mouse) | 5'-TAGAGGGACAAGTGGCGTTC-3' | 5'-CGCTGAGCCAGTCAGTGT-3' |
| RSAD2 (mouse) | 5-CTGTGCGCTGGAAGGTTT-3 | 5-ATTCAGGCACCAAACAGGAC-3 |
| Actin (mouse) | 5-GCCTTCCTTCTTGGGTATGG-3 | 5-AGCACTGTGTTGGCATAGAG-3 |
| mIFit1 (mouse) | 5-GAGAGTCAAGGCAGGTTTCT-3 | 5-TCTCACTTCCAAATCAGGTATGT-3 |
